# Maternal high-intensity interval training as a suitable approach for offspring’s heart protection in rat: evidence from oxidative stress and mitochondrial genes

**DOI:** 10.3389/fphys.2023.1117666

**Published:** 2023-05-23

**Authors:** Reihaneh Mohammadkhani, Alireza Komaki, Seyed Asaad Karimi, Mahdi Behzad, Shirin Heidarisasan, Iraj Salehi

**Affiliations:** ^1^ Neurophysiology Research Center, Hamadan University of Medical Sciences, Hamadan, Iran; ^2^ Department of Immunology, School of Medicine, Hamadan University of Medical Sciences, Hamadan, Iran; ^3^ Department of Biochemistry, School of Medicine, Hamadan University of Medical Sciences, Hamadan, Iran

**Keywords:** maternal exercise, offspring, high-intensity interval training, mitochondrial gene expression, oxidant-antioxidant status, elevated plus-maze, open-field

## Abstract

Considerable scientific evidence suggests that the intrauterine environment plays a crucial role in determining the long-term health of offspring. The present study aims to investigate the effects of high-intensity interval training in maternal rats before and during pregnancy on the antioxidant status, mitochondrial gene expression, and anxiety-like behavior of their offspring. A total of thirty-two female rats were assigned to four maternal groups based on the timing of exercise: before pregnancy, before and during pregnancy, during pregnancy, and sedentary. The female and male offspring were allocated to groups that matched their mothers’ exercise regimen. Anxiety-like behavior in the offspring was evaluated using the open-field and elevated plus-maze tests. Our findings indicate that maternal HIIT does not have any detrimental effect on the anxiety-related behavior of offspring. Also, maternal exercise before and during pregnancy could improve the general activity of the offspring. Furthermore, our results demonstrate that female offspring exhibit more locomotion activity than males. Besides, maternal HIIT leads to a reduction in the levels of TOS and MDA, while TAC levels increase, and significantly upregulate the gene expression of PGC1-α, NFR1, and NRF2 in both sexes in the heart. Therefore, our study suggests that maternal HIIT is a beneficial maternal behavior and serves as a cardioprotective agent to enhance the health of the next generations.

## Highlights

- Our findings establish that exercising during pregnancy does not need to be limited to low-intensity training, as high-intensity interval training can be deemed an optimal maternal intervention for safeguarding the heart health of future generations.
- Maternal high-intensity interval training can be considered an appropriate intervention before and during pregnancy to enhance the cardiac health of offspring by improving their antioxidant status and mitochondrial genes.

## 1 Introduction

A global health priority of the World Health Organization (WHO) is the fight against non-communicable diseases (NCDs), also known as chronic diseases that are the result of a combination of genetic, physiological, environmental, and behavioral factors. Toward the health promotion and prevention of chronic diseases, the WHO suggests early intervention in lifestyle to get the maximum benefit (World Health Organization, 2018). It is well established that regular exercise reduces the risk of lifestyle-related diseases including cardiovascular diseases, many metabolic disorders, and neurodegenerative diseases (Wen et al., 2011; Quiclet et al., 2017; Fiuza-Luces et al., 2018). Several studies have demonstrated the importance of maternal habits during preconception and conception periods on the subsequent development of offspring in later life (Aksu et al., 2012a; Woodman et al., 2018; Segabinazi et al., 2019; Woodman et al., 2020; Laker et al., 2021). A close relationship between maternal exercise and the promotion of offspring health has been shown in support of the importance of maternal intervention (Beeson et al., 2018; Chung et al., 2017; da Silva et al., 2016; Klein et al., 2018; Park et al., 2013; Mohammadkhani et al., 2020; Mangwiro et al., 2018).

The research of the last decade has highlighted the beneficial effects of maternal training on the health of offspring’s various tissue such as the brain (da Silva et al., 2016; Marcelino et al., 2013) and heart (Chung et al., 2017; Mohammadkhani et al., 2020). While the different animal models of exercise research have indicated maternal exercise as a positive maternal behavior during pregnancy, there are still many questions about optimum intensity, timing, and the dose of maternal exercise. High-intensity interval training (HIIT) has recently gained popularity as an effective time-efficient way to exercise, which involves short bouts of intense exercise alternated with recovery periods. The premise of using HIIT is that this intensity promotes better adaptations than moderate-intensity exercise via increased cellular stress (Kemi and Wisloff, 2010; Gibala et al., 2012; Freitas et al., 2018). There is considerable debate as to whether males and females respond similarly to HIIT and the notion of a sex difference during high-intensity exercise has gained momentum attention in the literature (Schmitz et al., 2020). Sex differences in response to HIIT have been attributed to females being more fatigue resistant and recovering faster than males (Ahn and Kim, 2020). In contrast, males have greater increases in cardiorespiratory fitness (American College of Sports Medicine, 2013; Islam et al., 2021). Despite an inter-individual variation in response to exercise (Eynon, 2017; Landen et al., 2019), very few studies address the between-sex variation in response to HIIT at the molecular level.

Mitochondria are a crucial part of cardiac cells, comprising one-third of the heart’s volume (Ingwall, 2002). High-intensity interval training can increase the number of functional mitochondria by upregulating genes like PGC1-α, NRF1, NRF2, and Tfam (Bishop et al., 2019). PGC-1α enhances the activity of NRF-1/2, promoting mtDNA replication, transcription, and protein formation, leading to cellular respiration (Kang and Li Ji, 2012). Tfam regulates mtDNA replication and transcription, and its promoter binds to NRF-1/2, allowing co-regulation between mitochondria and nuclear activation via the PGC-1α-NRF-1/2-Tfam pathway, promoting mitochondrial biogenesis (Dong et al., 2002; Qiu et al., 2019). Mitochondrial biogenesis was shown to improve in the heart of adult pups whose mothers exercised prior to and during pregnancy (Saiyin et al., 2019). Of interest, mitochondrial-related gene expression has been reported to depend on sex difference, such as the higher mitochondrial efficiency and less mitochondrial content observed in female rodents (Colom et al., 2007; Ventura-Clapier et al., 2017). Furthermore, there is evidence that sex differences affect oxidative stress generation (Tenkorang et al., 2018).

Although higher intensity training levels lead to more cellular reactions such as inflammation, increased oxidative stress, an increase in enzymatic antioxidant mechanisms, and a greater disturbance in the balance between oxidants and antioxidants, the mechanism underlying the impact of high-intensity interval maternal training on the oxidative stress and mitochondrial gene expression status of offspring remains poorly understood. A study has shown a connection between oxidative stress during pregnancy and negative outcomes for both the mother and the fetus, such as fetal growth restriction and preterm delivery (Ingwall, 2002). Furthermore, it has been observed that maternal training can mitigate oxidative stress and enhance the antioxidant capacity of the fetal heart (Schmitz et al., 2020).

One of the main questions in this study is to determine whether the high stress of high-intensity interval maternal training before and during pregnancy may play a protective role within the heart of the next-generation. We investigated the gender differences in measured parameters. Hence, if the intensity of maternal training during pregnancy leads to increased oxidant levels, adopting the exercise before pregnancy could mitigate the stress in the offspring. However, we sought to investigate maternal HIIT at different stages of pregnancy and pre-pregnancy on the oxidant (TOS and MDA), antioxidant (TAC, SOD, GPx) parameters, and mitochondrial genes expression (PGC1-α, NRF1, NRF2, and Tfam) in the heart of adult offspring. Additionally, to investigate the impact of maternal HIIT during pregnancy on the anxiety-like behavior of offspring, the stress level was measured by behavioral parameters (elevated plus-maze and open-field) in the male and female adult offspring.

## 2 Materials and methods

### 2.1 Exercise protocol paradigm

The present study took place at the Hamadan University of Medical Sciences (AEC: IR.UMSHA.REC.1397.528, Hamadan University of Medical Sciences, Hamadan, Iran) and under the guideline of the National Institutes of Health Guide for Care and Use of Laboratory Animals (NIH Publication no. 85-23, revised 1985). A total of 32 female Wistar rats, aged 8 weeks were purchased from Hamadan University of Medical Sciences and were maintained in the animal’s house with standard chow at 22°C ± 2°C on a 12-h light-dark cycle. Dams were divided into four maternal groups (n = 8) according to the periods of exercise: exercise before pregnancy (BP), exercise before and during pregnancy (BDP), exercise during pregnancy (DP), and sedentary or control (C). The animals were given a week to acclimate to the treadmill device (model: 2016 Tajhiz Gostar Omid Iranian, Iran) and become accustomed to running in it through physical handling.

**FIGURE 1:**
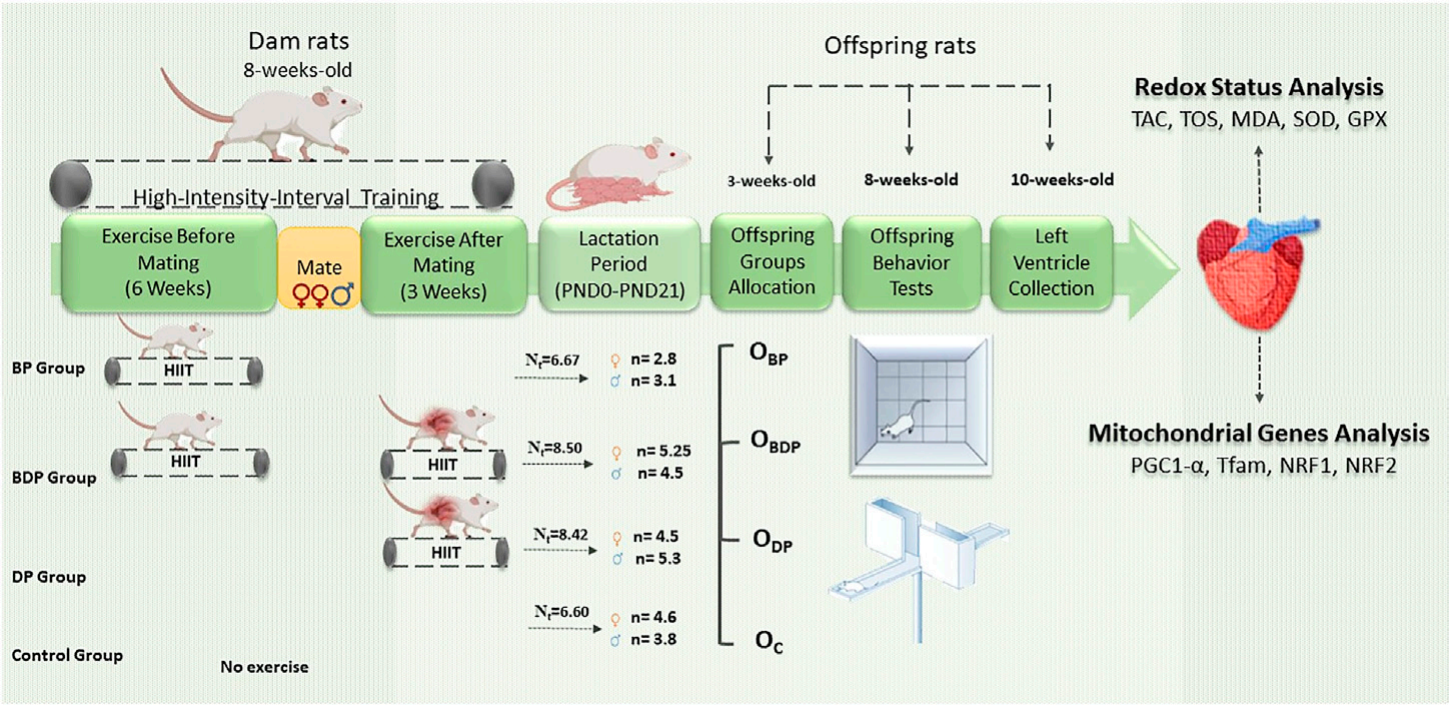
Schematic diagram of experimental design. Abbreviations: BP Group, dams who exercised before pregnancy; BDP Group, dams who exercised before and during pregnancy; DP Group, dams who exercised during pregnancy; Nt, the mean litter size of each mother groups. OBP, offspring born to dams who exercised before pregnancy (*n* = 6); OBDP, offspring born to dams who exercised both before and during pregnancy (*n* = 4); ODP, offspring born to dams who exercised during pregnancy (*n* = 6); OC, offspring born to control dams (*n* = 5).

The maternal exercise was first conducted for 6 weeks before pregnancy and then 3 weeks during pregnancy, during which dams were adapted to run according to their group. The speed of HIIT was determined by an exercise test that was performed prior to the main protocol (Välimäki et al., 2016). The exercise test detail is provided in our recent publication (Mohammadkhani et al., 2020). High-intensity interval training is defined as running on the treadmill with the speed of 85%–95% of VO2max for 3 min at 10° inclination which switching with active recovery (65% of VO2max) for 1 min at 0° inclination (Freitas et al., 2018; Mohammadkhani et al., 2020). The HIIT was performed 5 days per week. The mating in all groups (two females with one male) began after the completion of the first part of HIIT for 2 days. At the end of the protocol, dams that got pregnant were kept individually per cage to complete the birth. The litter size of all cages was randomly standardized to 4 pups (two female and two male) at postnatal day 2, so the remainder was placed into the dams which were unrelated to the present study. The pups were weaned at 3 weeks and then the female and male were allocated to offspring groups similar to their dam groups that included offspring born to dams who exercised before pregnancy (O_**BP**_), offspring born to dams who exercised before and during pregnancy (O_**BDP**_), offspring born to dams who exercised during pregnancy (O_**DP**_) and offspring born to control dams (O_**C**_). The number of offspring for each group is based on the number of pregnant mothers per group. Therefore, the number of rats in the O_**BP**_ group is six, in the O_**BDP**_ group is four, in the O_**DP**_ group is six, and in the O_**C**_ group is five. As they reach 8 weeks of age, plus-maze and the open-field test was evaluated in offspring groups. The offspring groups (10 weeks age) were anesthetized with Xylazine (3 mg/kg) and Ketamine (30 mg/kg), and the harvested tissue (left ventricle) was separated and washed with Phosphate-buffered saline (PBS); snap-frozen in liquid nitrogen and were stored at −80°C until further analysis. For an overview of the maternal and offspring groups as well as the experimental design refer to Figure 1.

### 2.2 Assessment of mitochondrial genes by mRNA analysis

The total RNA was isolated from frozen small left ventricle heart sections using RNA isolation of Kiazol protocol (Kiazist Life Sciences, Iran). RNA quality and quantity were assessed by gel electrophorus and Nano-Drop. Following, 2 μg of RNA were reverse transcribed to cDNA using the HyperScriptTM synthesis kit (GeneAll, Korea). The gene expression of PGC-1α, NRF1, NRF2, Tfam, and β-actin were then determined by real-time PCR using RealQ Plus 2X Master Mix Green (AMPLIQON, Denmark) and 20-μL reactions. The primer pairs used are provided in Supplementary Table S1 (upstream and downstream primers). β-actin as a housekeeping gene was utilized to normalize relative gene expression via the 2^−ΔCt^ method (Schmittgen and Livak, 2008).

### 2.3 Assessment of redox status analysis

The grinded tissues were lysed in the lysis buffer according to Kiazist kit (Kiazist Life Sciences, Iran) protocol—briefly, TAC (Total Antioxidant Capacity) and TOS (Total Oxidant Status): 200 μL PBS, MDA (Malondialdehyde): 300 μL MDA lysis buffer +3 μL BHT, SOD (Superoxide Dismutase) activity and GPx (Glutathione Peroxidases) activity: 200 μL PBS+ 8 μL protease inhibitor cocktail, then Samples were centrifuged at 20,000 × g for 10 min. The levels of TAC, TOS, MDA and SOD and GPx activity were measured by the Kiazist kit as per the manufacturer’s instructions. In brief, the TAC level was assessed using the CUPRAC (cupric reducing antioxidant capacity) spectrophotometric method. This method involves the reduction of cupric ions (Cu+2) to cuprous ions (Cu+1) in the presence of chromogen, which produces a color that is indicative of the presence of antioxidants. The final determination of the end-product is performed by colorimetry, and the resulting absorbance at 450 nm is measured. Also, The TOS level was determined by measuring the oxidation of ferrous ions to ferric ions, which produces a color in the presence of a chromogen. The resulting absorbance falls within the range of 580–550 nm. To assess lipid peroxidation, the quantity of thiobarbituric acid (TBA) reactive substances (TBARS) was measured as an index of malondialdehyde (MDA) production, and it was recorded at a wavelength of 532 nm. The measurement of SOD activity relies on the production of superoxide radicals by xanthine oxidase, which react with resazurin to generate detectable resorufin dye at 570 nm. The activity of GPx, based on the reduction of hydrogen peroxide to water accompanied by oxidation of glutathione, was determined by a colorimetric assay using a coupling reaction along with the glutathione reductase enzyme and its coenzyme, NADPH. The total protein concentration was determined by the BCA test and the values were normalized to the amount of protein per sample (50 mg).

### 2.4 Behavior test

#### 2.4.1 Open-field

The open-field test was conducted to assess the spontaneous locomotor activity of adult pups (at 9 weeks of age). Each pup was exposed to the center of the black wooden open-field box (40 cm × 50 cm×30 cm) and its locomotion activity was recorded for 10 min as the described method (Etaee et al., 2019). The apparatus was sprayed with 70% methanol between each test. The total distance (for analysis of locomotion), the number, and the time of entries into the central zone for analysis of anxiety-like behavior were automatically collected via the Smart Video Tracking System.

#### 2.4.2 Elevated plus-maze

The elevated plus-maze test was conducted to assess the anxiety-like behavior of offspring (at 9 weeks of age) that consisted of two open arms (50 × 10 cm), two closed arms (50 × 10 cm), and a central platform (10 × 10 cm) placed 50 cm above the floor. Each rat was placed in the central zone facing to close arm and was allowed to freely explore all parts of the maze for 10 min (Karimi et al., 2019). At the end of each test, the surface of the apparatus was sprayed with 70% methanol. The following behavioral parameters were automatically recorded: duration and number of entries into the open arms, closed arms, and total distance travel. In addition, the percentage of duration (time on the open arms/total time on the open and close arms) and open arms entries (open arms entries/total open and close arms entries), the percentage of close arms entries (close arm entries/total open and close arms entries) was calculated. Behavioral parameters were analyzed automatically using Smart Video Tracking System. Anxiety reduction in the plus-maze is indicated by an increase in the proportion of time spent in the open arms and an increase in the proportion of entries into the open arms.

### 2.5 Statistical analysis

Data were processed using a statistical package of GraphPad Prism^®^ 9. To evaluate the test distributions, all data were analyzed by the Shapiro-Wilk normality test and then significant differences, the parametric data were analyzed by One-way and Two-way ANOVA, and the non-parametric data were analyzed by the Kruskal-Wallis test. Two-way ANOVA was conducted with offspring groups serving as the column factor and gender differences as the row factor. Post-hoc tests were performed using Dunnett (for the main effect of exercise groups) and Sidak’s multiple comparisons (for the main effect of gender difference). All the values are presented as the mean ± SD. A probability of *p* values < 0.05 was considered to be statistically significant.

## 3 Results

As this study was a part of a larger research project, information on the characteristics of the pups is presented in another article (Mohammadkhani et al., 2020). Here, we will focus on the effects of maternal HIIT on the stress-oxidative and mitochondrial genes expression of the adult offspring.

### 3.1 Litter characteristics (descriptive statistics)

**FIGURE 2:**
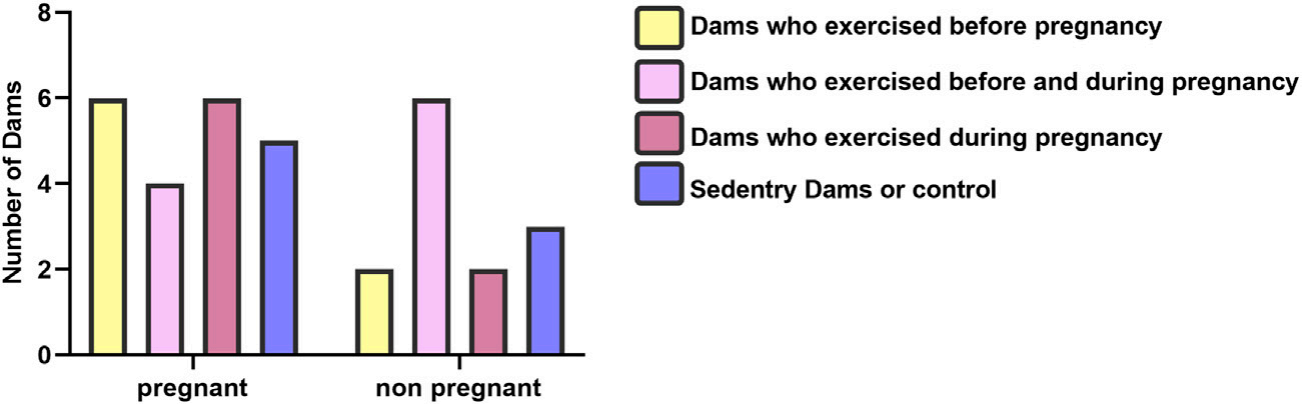
Litter characteristics of dam groups.

Although some animals did not become pregnant during the mating process, all rats in their respective groups completed the exercise protocol. The present study began with 32 rats. A total of 21 rats became pregnant, which include six rats who became pregnant in the BP group, four rats who became pregnant in the BDP group, six rats who became pregnant in the DP group, and five rats became pregnant in the BP group, in detail. The results of the Kruskal-Wallis test showed no significant (*p* = 0.36) difference between pregnancy rates among mother groups (Figure 2). There was not statistically significant in the sex distribution (*p* = 0.10) and birth weight (*p* = 0.246) of offspring groups as previously published (Mohammadkhani et al., 2020).

### 3.2 Maternal high-intensity interval training improved offspring heart’s redox status

**FIGURE 3:**
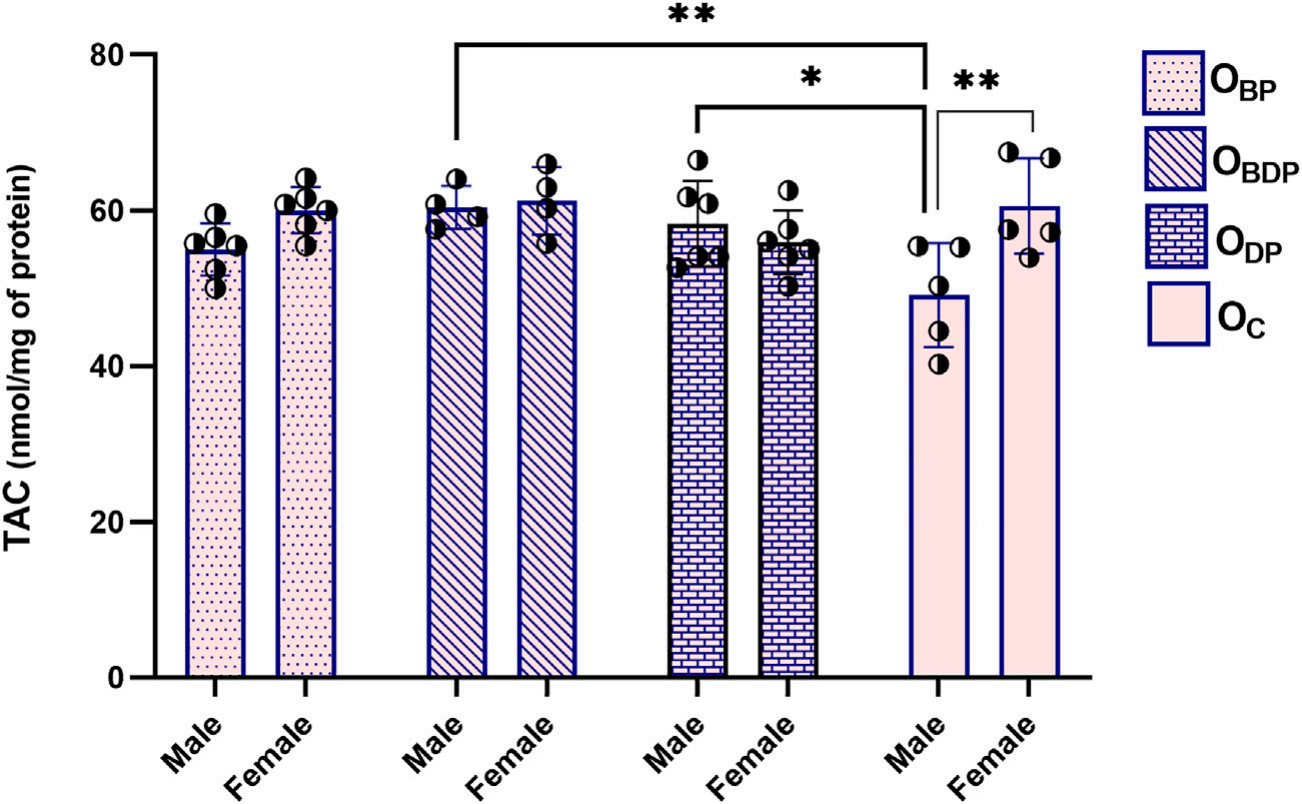
Maternal HIIT increases total antioxidant capacity of male offspring. Abbreviations: OBP, offspring born to dams who exercised before pregnancy (*n* = 6); OBDP, offspring born to dams who exercised both before and during pregnancy (*n* = 4); ODP, offspring born to dams who exercised during pregnancy (*n* = 6); OC, offspring born to control dams (*n* = 5). *p < 0.05, **p < 0.01 vs. OC, Data are Mean ± SD. TAC, total antioxidant capacity.

Figure 3 displays the two-way ANOVA result of TAC, which indicates a significant difference (F1,34 = 7, *p* = 0.022) between males and females in the control group as determined by Sidak’s multiple comparisons tests. In addition, within the male offspring, the TAC levels in the O_**BDP**_ and O_**DP**_ groups were significantly higher (F1,17 = 4.8, *p* = 0.008, *p* = 0.017) than those in the control group. Conversely, there was no significant difference (*p* = 0.223) between the female offspring groups, as indicated by Dunnett’s multiple comparisons tests.

**FIGURE 4:**
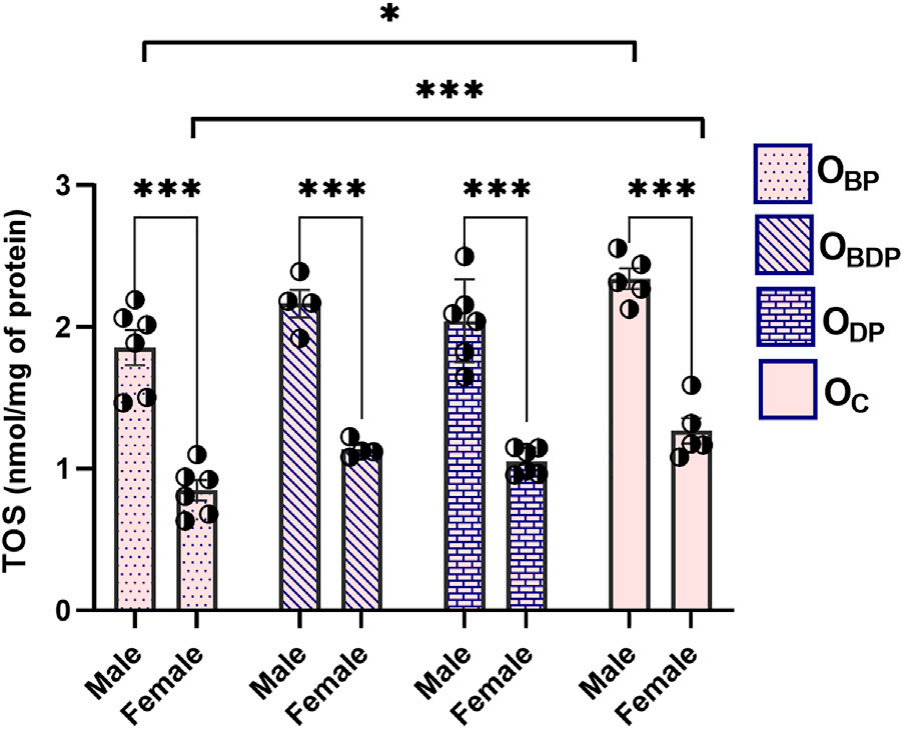
Maternal HIIT decreases total oxidant status of offspring. Abbreviations: OBP, offspring born to dams who exercised before pregnancy (*n* = 6); OBDP, offspring born to dams who exercised both before and during pregnancy (*n* = 4); ODP, offspring born to dams who exercised during pregnancy (*n* = 6); OC, offspring born to control dams (*n* = 5). *p < 0.05, ***p < 0.001 vs. OC, Data are Mean ± SD. TOS, total oxidant status.

The analysis of TOS in Figure 4 revealed a significant difference between all male and female offspring groups (F1,34 = 247, *p* < 0.001), as indicated by Sidak’s multiple comparisons tests. Maternal exercise before pregnancy could significantly reduce TOS levels in both male (F1,17 = 3.5, *p* = 0.015) and female (F1,17 = 7.7, *p* = 0.000) offspring.

**FIGURE 5:**
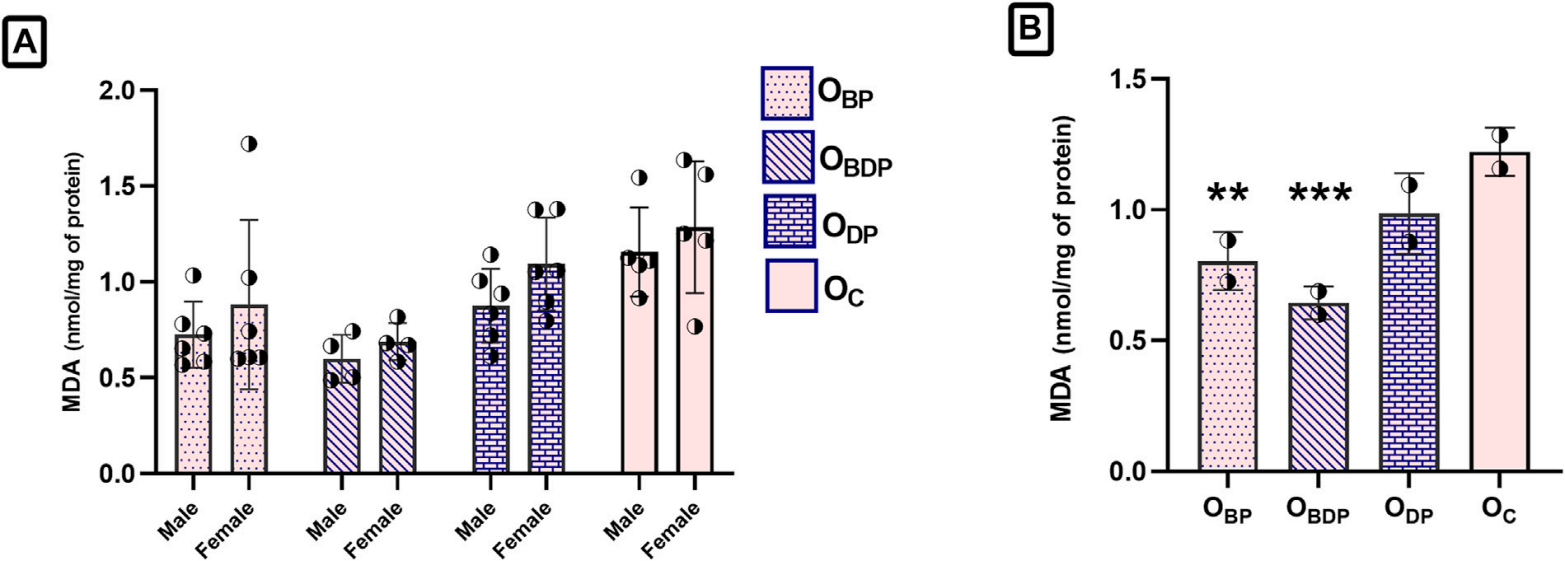
Maternal HIIT decreases the malondialdehyde of offspring. **(A)** Sex difference in the MDA of offspring group, **(B)** The one-way ANOVA of MDA in the combined male and female offspring groups. Abbreviations: OBP, offspring born to dams who exercised before pregnancy (*n* = 6); OBDP, offspring born to dams who exercised both before and during pregnancy (*n* = 4); ODP, offspring born to dams who exercised during pregnancy (*n* = 6); OC, offspring born to control dams (*n* = 5). **p < 0.01, ***p < 0.001 vs. OC, Data are Mean ± SD. MDA, malondialdehyde.

As shown in Figure 5A, the ANOVA analysis of MDA revealed no significant sex differences (F1,34 = 3.23, *p* = 0.08). As a result, the data for male and female offspring were combined and analyzed to examine the main effects of pup groups (Figure 5B). The study found that maternal exercise before pregnancy, as well as before and during pregnancy, significantly reduced lipid peroxidation levels in both male and female offspring compared to the control group. This effect was statistically significant using Dunnett’s multiple comparisons tests (F3,34 = 8, *p* < 0.001).

**FIGURE 6:**
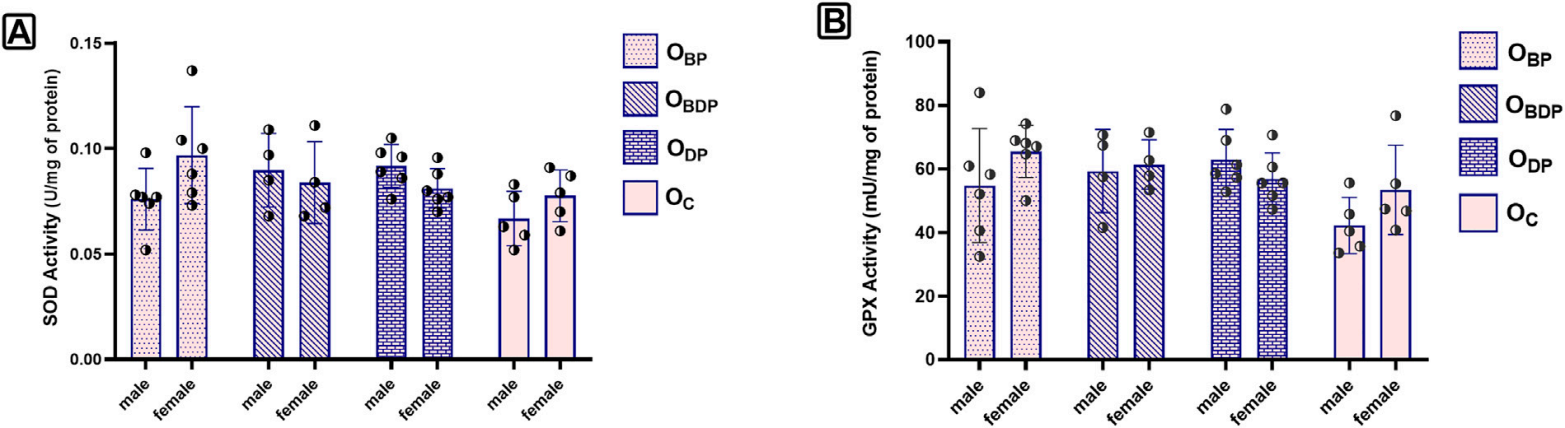
The levels of SOD and GPX activity of offspring. **(A)** The two-way ANOVA of SOD, **(B)** The two-way ANOVA of GPx, Abbreviations: OBP, offspring born to dams who exercised before pregnancy (n = 6); OBDP, offspring born to dams who exercised both before and during pregnancy (n = 4); ODP, offspring born to dams who exercised during pregnancy (n = 6); OC, offspring born to control dams (n = 5)., Data are Mean ± SD. SOD, Super Oxide Dismutase and GPx, Glutathione Peroxidases.

Despite the increase in the levels of both SOD and GPx activity observed (Figure 6), the statistical analysis using a two-way ANOVA indicated that this increase was not significant for any of the outcome variables (*p* > 0.05).

### 3.3 Maternal HIIT alters offspring heart mitochondrial gene expression

Gene expression associated with mitochondrial biogenesis is presented in Table 1. Maternal HIIT before and during pregnancy significantly affected the gene expression of offspring in the heart tissues. The result of two-way ANOVA indicated maternal HIIT before and during pregnancy or only during pregnancy significantly increased the genes expression of PGC1-α (F3,34 = 11, *p* < 0.001), NRF1 (F3,34 = 8, *p* < 0.001), NRF2 (F3,34 = 19, *p* < 0.001) and Tfam (F3,34 = 3, *p* = 0.04). By considering the results of Sidak’s multiple comparisons tests for gender differences, a significant difference was observed in the gene expression of Tfam between male and female offspring (F1,34 = 6, *p* = 0.02).

**TABLE 1:** Biogenesis mitochondria genes expression of male and female pups in the Heart tissue.

Gene	Sex	Offspring born to dams who exercised before pregnancy (n = 6)	Offspring born to dams who exercised before and during pregnancy (n = 4)	Offspring born to dams who exercised during pregnancy (n = 6)	Offspring born to control dams (n = 5)
PGC1-α	M	1.00	**1.85** ^*^	1.59	0.99
	F	1.11	**1.96** ^*^	**1.93** ^*^	0.92
Nrf1	M	0.91	1.30	**1.58** ^*^	0.88
	F	1.16	**1.37**	**1.88** ^*^	1.17
Nrf2	M	1.31	**2.37** ^**^	**2.61** ^***^	0.92
	F	1.02	1.71	**1.92** ^*^	1.03
Tfam	M	0.96	1.25	**1.05** ^**##**^	0.95
	F	1.23	1.68	**2.20** ^*^	0.95

*p < 0.05.

**p < 0.01.

***p < 0.001 vs. control.

^*##*^ *p* < 0.01 between male and female of each group. Data are means (SD). M, male. F, female. *p* values are bold where significant.

### 3.4 Maternal HIIT reduces offspring anxiety-like behavior

#### 3.4.1 Open-field test

**FIGURE 7:**
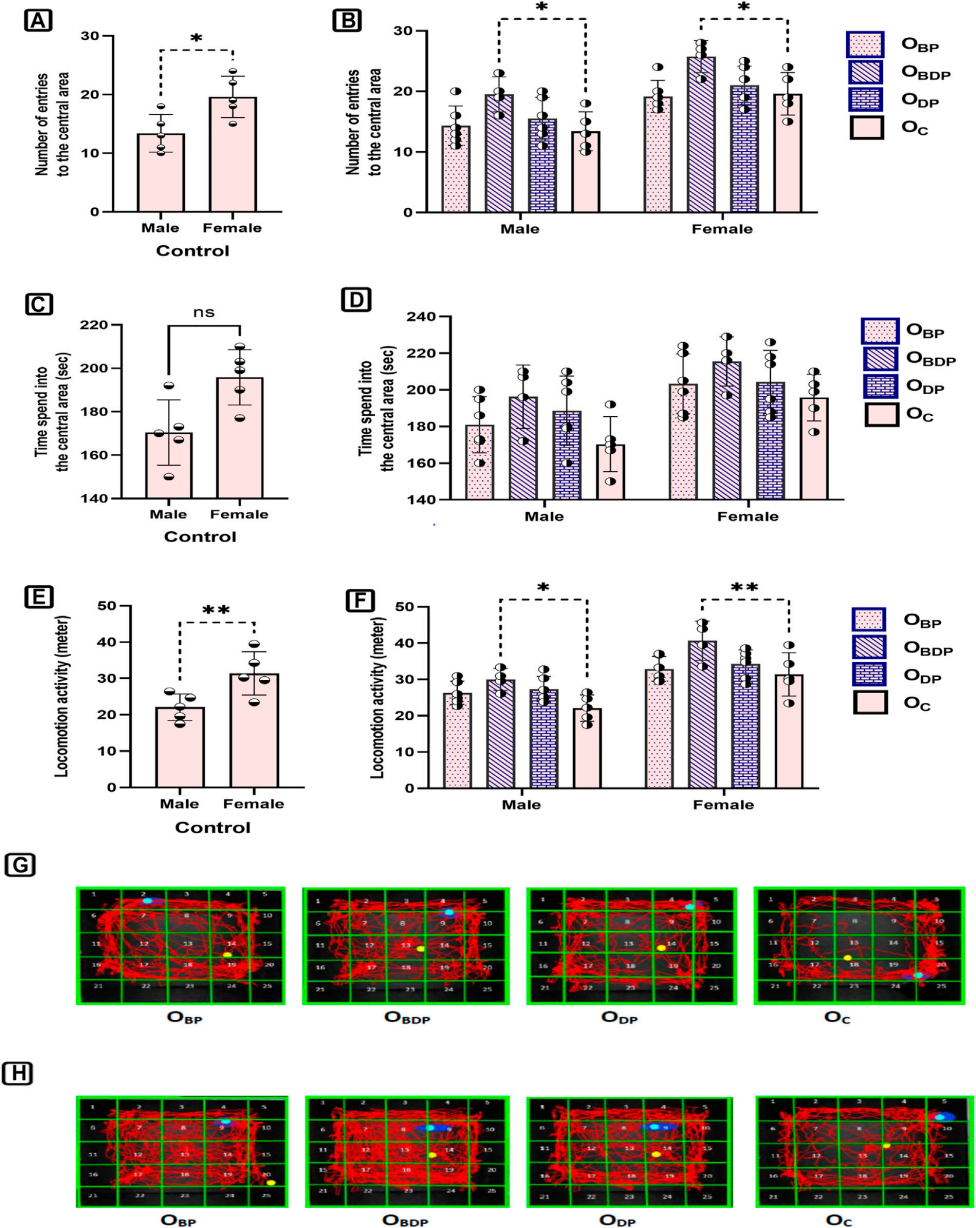
The analysis of open field parameters of offspring. **(A,B)** The number of entries into the center zone, **(C,D)** The time of entries into the center zone, **(E,F)** The total distance, **(G)** Representative figures of a male in different study groups, **(H)** Representative figures of a female in different study groups. Abbreviations: OBP, offspring born to dams who exercised before pregnancy (n = 6); OBDP, offspring born to dams who exercised both before and during pregnancy (n = 4); ODP, offspring born to dams who exercised during pregnancy (n = 6); OC, offspring born to control dams (n = 5). *p < 0.05, **p < 0.01 vs. OC, Data are Mean ± SD.

To investigate sex differences in the open-field test (Figures 7A, C, E), a two-way ANOVA was performed, which revealed significant sex differences for two behavioral parameters in the open-field box. Sidak’s multiple comparisons tests were used to examine the effects of sex. The females made a greater number of entries into the central area (F1,34 = 34, *p* < 0.001), and traveled a longer distance than the males (F1,34 = 43.53, *p* < 0.0001). The current study examined the effect of maternal HIIT on the anxiety-like behavior of offspring (Figures 7B, D, F). Results showed that maternal training before and during pregnancy led to male and female offspring exhibiting more entries to the central area and greater locomotion activity compared to other groups. This indicates a reduction in anxiety-like behavior in this group of offspring.

#### 3.4.2 Elevated plus-maze

**FIGURE 8:**
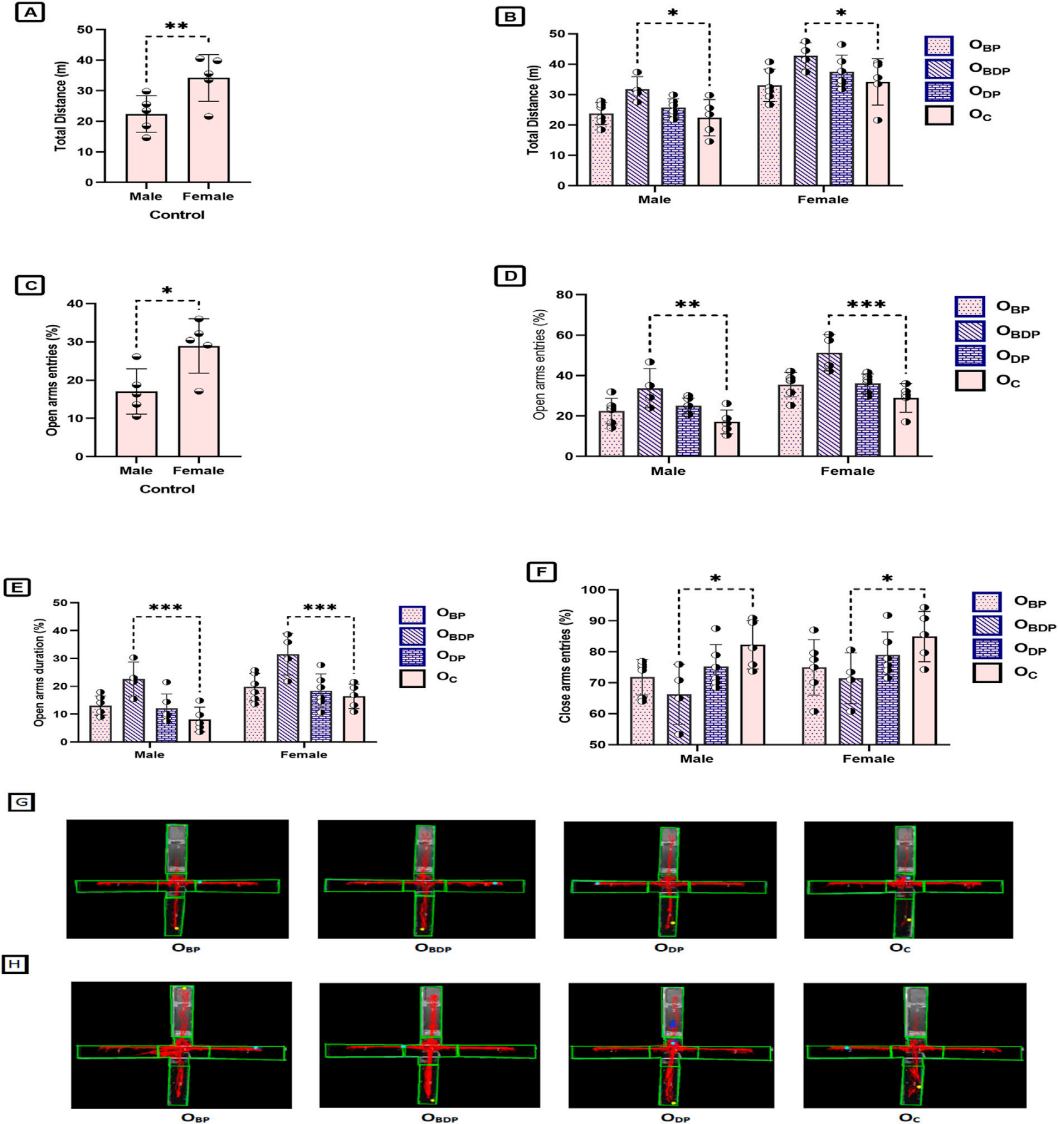
The analysis of elevated plus-maze parameters of offspring. **(A,B)**. The total distance, **(C,D)** The number of open arms entries, **(E)** Time spent on the open arms, **(F)** The number of close arms entries, **(G)** Representative figures of a male in the study groups, **(H)** Representative figures of a female in the study groups. Abbreviations: OBP, offspring born to dams who exercised before pregnancy (n = 6); OBDP, offspring born to dams who exercised both before and during pregnancy (n = 4); ODP, offspring born to dams who exercised during pregnancy (n = 6); OC, offspring born to control dams (n = 5). *p < 0.05, **p < 0.01, ***p < 0.001 vs. OC. Data are Mean ± SD.

The present study used the Elevated plus-maze to examine the impact of maternal HIIT on the anxiety-like behavior of offspring (as shown in Figures 8A–D). The results indicated that male and female offspring of mothers who underwent HIIT before and during pregnancy showed increased entries and duration in the open arms (F1,34 = 43, *p* < 0.001), reduced entries in the closed arms (F1,34 = 5.86, *p* = 0.002), and greater distance traveled (F1,34 = 6.02, *p* = 0.002) compared to other groups. This suggests that maternal exercise can reduce anxiety-like behavior in offspring. Furthermore, analysis using a two-way ANOVA followed by Sidak’s *post hoc* multiple comparisons revealed significant sex differences in two behavioral parameters in the Elevated plus-maze test, as demonstrated in Figures 8E, F The females made more entries into the open arms (F1,34 = 43.41, *p* < 0.0001), and traveled longer distance than the males (F1,34 = 47.57, *p* < 0.0001).

## 4 Discussion

The lifelong health benefits of moderate-intensity maternal exercise during pregnancy are evident (Bahls et al., 2014; Agbas, 2015; Blaize et al., 2015; August et al., 2020). High-intensity training during pregnancy for active mothers without exercise restrictions is probably beneficial (Pivarnik et al., 2016). We have previously demonstrated optimal periods for the high-intensity interval activity of mothers (before and during pregnancy) and the usefulness of maternal HIIT on the cardiac genes of offspring (Mohammadkhani et al., 2020). Given that high-intensity interval training has been shown to provide greater health benefits in a shorter amount of time (Jiménez-Maldonado et al., 2018; Bishop et al., 2019) *and promotes increased cellular stress adaptations compared to moderate intensities, we have expanded our investigation to determine whether high-intensity interval training for pregnant mothers can alter the redox hemostasis and genes involved in mitochondrial biogenesis in the heart tissue of offspring. Furthermore, we evaluated the anxiety behavior parameters and sex-specific responses of the offspring to these periods of maternal exercise. Studies have demonstrated that lifestyle interventions can improve health outcomes by decreasing mitochondrial dysfunction and oxidative stress* (Marques-Aleixo et al., 2012; Bhatti et al., 2017; Freitas et al., 2018). Exercise appears to increase reactive oxygen species, which can result in the enhancement of the antioxidant defense system. Interventions that increase cardiac antioxidant defense can promote a healthy heart (Valaei et al., 2021).

Interestingly, our findings indicate that performing high-intensity interval training (HIIT) during pregnancy not only has no negative impact on the offspring’s oxidant biomarkers, but also enhances their antioxidant status and mitochondrial gene expression in the heart, for both male and female offspring. Furthermore, our study found that early-life stress induced by maternal training did not have any adverse influence on the anxiety factors of the offspring. In fact, maternal high-intensity interval training (HIIT) before and during pregnancy may actually reduce anxiety parameters and improve the general activity of the next-generation, as we hypothesized. One noteworthy finding of our study is that maternal exercise promotes the overall activity of the offspring, consistent with previous research (Eclarinal et al., 2016; Knight et al., 2021). Considering that physical inactivity is linked to an increased risk of chronic heart disease in adulthood (Anderson and Durstine, 2019), our study suggests that the significant increase in total traveled distance by offspring born to mothers who exercised before and during pregnancy could have a remarkable impact on preventing cardiovascular diseases related to inactivity. As expected, the female offspring displayed more locomotion activity than the male offspring in both behavioral tests. The present study revealed that female offspring of Wistar rats display less anxiety-like behavior than male offspring in the open field and elevated plus maze tests, which is consistent with earlier literature (Knight et al., 2021). Based on our results, it can be concluded that maternal HIIT before and during pregnancy is an efficient factor in reducing the anxiety-like behavior of offspring. However, contrary to our findings, other studies have shown that maternal swimming did not alter the results of the offspring’s open field test (Klein et al., 2019). A study indicated that mothers who exercise during pregnancy give birth to offspring with more explorative behavior and lower anxiety levels (Aksu et al., 2012b). Therefore, the different results may be attributed to the different maternal exercise models used.

Excessive stress during pregnancy can initiate a cascade of molecular events, endangering the health of the offspring. This can lead to oxidative stress, which damages cellular components of offspring such as proteins, lipids, and DNA, and ultimately results in mitochondrial dysfunction, compromised myocardial structure and metabolism, decreased cardiac performance, and increased susceptibility to adult ischemic injury (Giussani et al., 2012; Manti et al., 2020; Yang et al., 2020). Maternal physical activity has been shown to have several positive effects on the offspring’s cardiovascular system, such as promoting proper cardiogenesis, reducing the levels of reactive oxygen species (ROS) in vascular smooth muscle, increasing mitochondrial enzymatic activity and ATP production, and decreasing hydrogen peroxide levels in fetal mouse hearts (Fukai et al., 2000; Chung et al., 2017; Saiyin et al., 2019). Evidence suggests that maternal exercise decreases oxidative stress in the fetal heart and maintains redox balance in the hearts of offspring affected by pre-gestational diabetes (Saiyin et al., 2019). Additionally, studies have shown that a different kind of moderate-intensity maternal training has a beneficial effect on the redox hemostasis of pups (Marcelino et al., 2013; Park et al., 2013; August et al., 2020). However, only a limited number of studies have investigated the effects of maternal high-intensity interval during pregnancy (Songstad et al., 2015; Pivarnik et al., 2016). The most striking finding of the present study is the introduction of HIIT during pregnancy as an acceptable maternal exercise. This may improve the ability to maintain redox balance in the adult offspring. Despite the ineffectiveness of maternal HIIT on the levels of TAC and MDA content in the placenta and fetal heart as demonstrated by previous research (Pivarnik et al., 2016), our findings indicate that maternal HIIT can alter the levels of TAC (in male offspring only), TOS, and MDA in the hearts of adult offspring. SOD and GPx are endogenous antioxidants that play a crucial role in maintaining the delicate balance between oxidants and antioxidants in the body. Previous research has demonstrated that maternal exercise during pregnancy can enhance the offspring’s ability to maintain redox balance by increasing the levels of endogenous antioxidants, including SOD and GPx (Chung et al., 2017; Kusuyama, 2022). However, the findings of the current investigation propose that maternal high-intensity interval training did not result in a significant increase in the enzymatic antioxidant activity of offspring, which is consistent with earlier research on maternal HIIT (Songstad et al., 2015). A possible reason for the absence of significance in the observed increase in enzymatic antioxidant activity, such as SOD and GPx, could be attributed to the sample size, as a larger sample size may yield statistical significance. The noteworthy insights regarding high-intensity interval training are derived from the levels of TOS and MDA observed in the offspring groups. It is interesting to note that maternal adaptation to high-intensity exercise before pregnancy results in a reduction of the oxidant status of adult offspring. The improved oxidant-antioxidant status could be attributed to various changes, and one plausible explanation is the increased physical activity of the offspring. This is supported by the observed increase in the locomotion activity of male and female offspring during the open-field and elevated plus-maze tests.

The normal development of mitochondrial function can be considered a worthy process that ensures the body’s health. The heart, due to its high concentration of mitochondria, plays a crucial role in maintaining mitochondrial quality and redox homeostasis. Balances between oxidative and antioxidative processes are a common mediator of cardiac cell health. An animal study has shown that maternal exercise during pregnancy can improve mitochondrial function in the fetal heart of offspring (Chung et al., 2017). As expected, maternal HIIT upregulated genes associated with mitochondrial biogenesis (PGC1-α, Nrf1, Nrf2, and Tfam in Table 1) in the hearts of offspring. In the current study, the gene expression of PGC1-α, NRF1, and NRF2 did not differ between the sexes, but there was a sex-related difference in Tfam expression. Our results demonstrate that maternal HIIT can induce a positive mitochondrial phenotype in offspring. This highlights the importance of maternal activity in improving the health of offspring in later life. These findings are among the first concerning maternal high-intensity interval exercise training, and further studies are needed to investigate its effects on offspring. We did not directly measure the quantity or mass of mitochondria in the hearts of the offspring, which is one of the study’s limitations. Direct measurement of mitochondrial content would offer more conclusive proof of the positive effects of maternal HIIT on offspring’s mitochondrial function, even though upregulation of genes linked to mitochondrial biogenesis indicates an increase in mitochondrial number/mass.

In conclusion, we have been able to detect that the stress of maternal high-intensity interval training during pregnancy did not increase the levels of TOS and MDA in offspring. We simultaneously measured redox hemostasis and genes’ expression associated with mitochondrial biogenesis at the left ventricle tissue and concluded that maternal HIIT before and during pregnancy significantly improved the redox status and genes’ expression of mitochondrial biogenesis of offspring. Overall, we have confirmed that female offspring were more active and were less anxious than male offspring. Also, there were no significant differences in the results obtained from mitochondrial biogenesis factors of offspring based on sex. For the first time to our knowledge, this study also provides a piece of evidence that maternal HIIT health consequences persist in adulthood. Hence, due to the lifelong health benefits of maternal HIIT on the next-generation, this model of exercise is advisable before and during pregnancy. The fact that maternal exercise before and during pregnancy can bestow some health benefits to offspring is valuable, especially if these results could translate to humans.

## Data Availability

The original contributions presented in the study are included in the article/Supplementary Materials, further inquiries can be directed to the corresponding author.
