# Supplementary material for: Maternal high-intensity interval training as a suitable approach for offspring’s heart protection in rat: evidence from oxidative stress and mitochondrial genes
Source: Front Physiol. 2023 May 23;14:1117666. doi: 10.3389/fphys.2023.1117666 (PMC10242028; doi:10.3389/fphys.2023.1117666)
Supplement: Supplementary file 1 [file Table1.DOCX]

# Supplementary Material

**S1 Table: Conditions and Sequence of primers**

| gene | sequence | | PCR PRODUCT SIZE | AMPLICON T_m_ |
| --- | --- | --- | --- | --- |
| PGC1-α | Forward | cag acc tag att caa act cag acg | 70 | 60^◦^C |
|  | Reverse | aaa tcc aga gag tca tac ttg ctc |  |  |
| NRF1 | Forward | tag ccc atc tcg tac cat cac | 186 | 60^◦^C |
|  | Reverse | ttt gtt cca cct ctc cat cag |  |  |
| NRF2 | Forward | gtc gct tgc cct gga tat tc | 183 | 60^◦^C |
|  | Reverse | tcc gta tta aga cac tgt aac tcg |  |  |
| Tfam | Forward | aag cac aaa tca aga gga gag aat | 284 | 60^◦^C |
|  | Reverse | cac act gcg acg gat gag at |  |  |
| b-actin | Forward | atc agc aag cag gag tac gat | 94 | 60^◦^C |
|  | Reverse | aaa ggg tgt aaa acg cag ctc |  |  |
